# Effects of Combined Therapy with SGLT2i and GLP-1RAs on Atrial Fibrillation Recurrence After Catheter Ablation in Diabetic Cohorts: One-Year Outcomes from Continuous Monitoring

**DOI:** 10.3390/ijms26199285

**Published:** 2025-09-23

**Authors:** Celestino Sardu, Raffaele Marfella

**Affiliations:** 1 Department of Advanced Medical and Surgical Sciences, University of Campania Luigi Vanvitelli, 80138 Naples, Italy; raffaele.marfella@unicampania.it; 2 Pollution and Cardiovascular Diseases Research Centre, University of Campania Luigi Vanvitelli, 80138 Naples, Italy

**Keywords:** atrial fibrillation, diabetes mellitus type 2, GLP-1Ras, SGLT2i

## Abstract

To evaluate the effect of sodium–glucose cotransporter-2 inhibitors (SGLT2i), glucagon-like peptide-1 receptor agonists (GLP-1RAs), and their combination on atrial fibrillation (AF) recurrence after catheter ablation in patients with type 2 diabetes mellitus (T2DM). In a prospective cohort study, patients with T2DM undergoing AF ablation were stratified into three groups: SGLT2i-users, GLP-1RAs-users, and combined SGLT2i/GLP-1RAs users. Diabetics under SGLT2i/GLP-1RAs therapy had worse glycemic control (HbA1c > 7%). AF recurrence was assessed over 12 months using implantable continuous monitoring (ICM). Secondary outcomes included the inflammatory/oxidative stress markers measured at the 12-month follow-up. At the follow-up end, patients treated with SGLT2i/GLP-1RAs versus monotherapy patients showed significantly lower AF recurrence and serum inflammatory/oxidative stress markers, despite having higher HbA1c levels (*p* < 0.05). Combined SGLT2i/GLP-1RAs therapy reduced AF recurrence following catheter ablation and inflammatory/oxidative stress in T2DM patients.

## 1. Introduction

Type 2 diabetes mellitus (T2DM) and worse glycemic control could increase atrial fibrillation (AF) recurrence post-catheter ablation [1]. Hyperglycemia and elevated levels of hemoglobin A1c (HbA1c) are both strongly associated with an increased rate of AF recurrence post-ablation in T2DM patients, via over-inflammation/oxidative stress [2]. In this context, new anti-T2DM therapies, as the sodium–glucose-transporter 2 inhibitors (SGLT2i) and Glucagon-Like Peptide-1 Receptor Agonists (GLP-1RAs), have been safely used to reduce AF recurrence post-catheter ablation in T2DM cohorts [3,4]. Intriguingly, SGLT2i could reduce AF recurrence after catheter ablation via anti-inflammatory/oxidative effects and favoring the myocardial substrate modulation [5]. These effects are linked to the amelioration of cardiac electrophysiological properties in T2DM patients and lead to the best clinical outcomes [5]. Similarly, GLP-1 RAs reduce the AF recurrence following ablation via anti-inflammatory/oxidative effects and significant weight loss [4,6]. Therefore, assuming the over-inflammation/oxidative stress and the worse glycemic control as the main causes of AF recurrence post ablative approach, we might speculate that adding the SGLT2i to chronic therapy with GLP-1RAs in patients with worse glycemic control (Hb1Ac > 7 mg/dl) might result in the amelioration of glycemic control, and then in the significant reduction in inflammatory/oxidative stress in these patients. These effects could consequently reduce the AF recurrence post-ablation. On the other hand, we have no conclusive data about the effects played by SGLT2i added to GLP-1RAs on the AF recurrence post-ablation in T2DM patients, and particularly in those with over-inflammation/oxidative stress and worse glycemic control. Furthermore, in the current study, we investigated the relationship between glycemic control, inflammatory/oxidative stress, and AF recurrence after ablation in T2DM patients divided into patients under SGLT2i therapy (SGLT2i-users) vs. patients under GLP-1RAs therapy (GLP-1RAs-users) vs. patients under combined therapy with SGLT2i and GLP-1RAs (GLP-1RAs/SGLT2i-users). The AF recurrence post-catheter ablation was evaluated at 1 year of follow-up by implantable continuous monitoring (ICM) devices. Secondly, we evaluated the inflammatory/oxidative stress burden in the three study cohorts of T2DM patients at the follow-up end.

## 2. Results

In Table 1 we reported the characteristics of study cohorts at baseline and at 1 year of follow-up in the three study cohorts.

At baseline, the study cohorts did not differ in demographic characteristics, comorbidities, laboratory values, and drug therapy (*p* > 0.05). The patients under combined GLP-1RAs/SGLT-2i therapy vs. SGLT2i-users and vs. GLP-1RAs-users had higher glycemia and Hb1Ac values, and a lower glomerular filtration rate at baseline (*p* < 0.05). The cohorts did not differ for diabetes duration: 6.5 ± 3.8 vs. 5.9 ± 4.1 vs. 6.9 ± 4.6 years, by comparing group 1 vs. group 2 vs. group 3 (*p* > 0.05).

The median AF time was approximately 18 months (IQR 12–30) in the SGLT2i group vs. 17 months (IQR 11–28) in the GLP1-RA group vs. 19 months (IQR 12–29) in the combined SGLT2i/GLP-1RAs therapy group (*p* > 0.05). In the SGLT2i group, 179 patients (81.4%) underwent radiofrequency (RF) ablation and 41 (18.6%) underwent pulsed field ablation (PFA), while in the GLP1-RA group, RF was performed in 154 patients (79.8%) and PFA in 39 (20.2%), and 55 patients (79.7%) underwent RF ablation and 14 (20.3%) PFA in the combined GLP1-RA/SGLT2i group (*p* > 0.05). Thus, the distribution of ablation modality did not differ significantly among the three groups.

At follow-up end, the patients under combined GLP-1RAs/SGLT2i therapy vs. SGLT2i-users and vs. GLP-1RAs-users had a lower heart rate and a lower percentage of them was under rate flecainide therapy (*p* < 0.05), despite worse glycemic control (Table 1). The primary outcome was achieved in 12 (17.4%) of patients under combined GLP-1RAs/SGLT2i therapy, compared to 64 (29.1%) SGLT2i-users and 57 (28.0%) GLP-1RAs Users (*p* < 0.05). In component-level analyses, AF recurrence was the most frequent event across all groups, whereas cardioversion and repeat ablation were less common. Specifically, AF recurrence occurred in 27.3% of the SGLT2i group (60/220), 30.1% of the GLP1-RA group (58/193), and 27.5% of the combined therapy group (19/69) (*p* > 0.05). Cardioversion was required in 10.9% (24/220), 13.0% (25/193), and 11.6% (8/69), respectively (*p* > 0.05), and redo ablation in 8.2% (18/220), 8.8% (17/193), and 8.7% (6/69) (*p* > 0.05). The overall primary composite endpoint occurred in 31.8%, 34.2%, and 33.3% of patients, respectively (*p* > 0.05).

**Figure 1:**
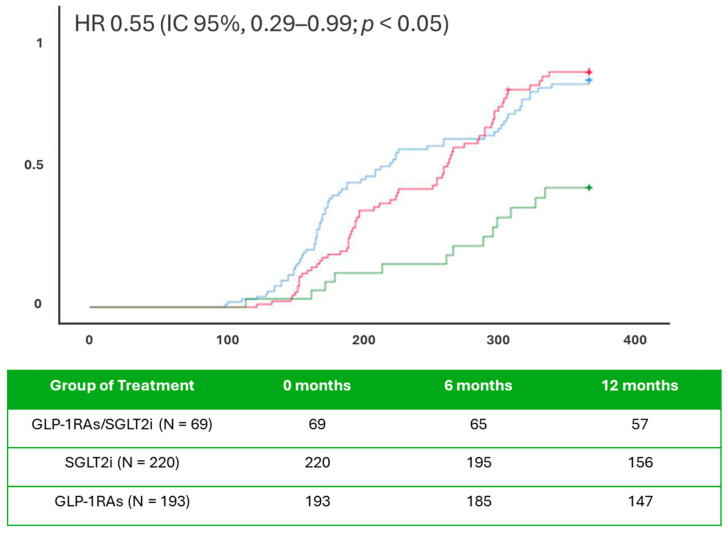
In the upper part of figure, the Unadjusted Kaplan curves for cumulative risk of having AF recurrence at 365 days of follow-up; in the lower part of figure, under the Kaplan curves, the number at risk for each cohort of study at 0, 6, and 12 months of follow-up. Blue color: SGLT2i-users, red color: GLP-1RAs users, green color: SGLT2i/GLP-1RAs users. GLP-1RAs: Glucagon-Like Peptide-1 Receptor Agonists; SGLT2i: sodium–glucose transporter 2 inhibitors; SGLT2i/GLP-1RAs: combined therapy with sodium–glucose transporter 2 inhibitors and Glucagon-Like Peptide-1 Receptor Agonists; HR: Hazard ratio; CI: confidence of interval; statistically significant (*p* < 0.05).

**Figure 2:**
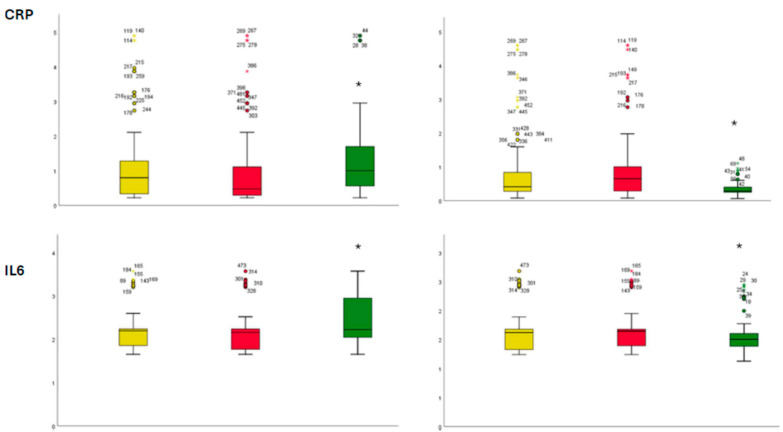

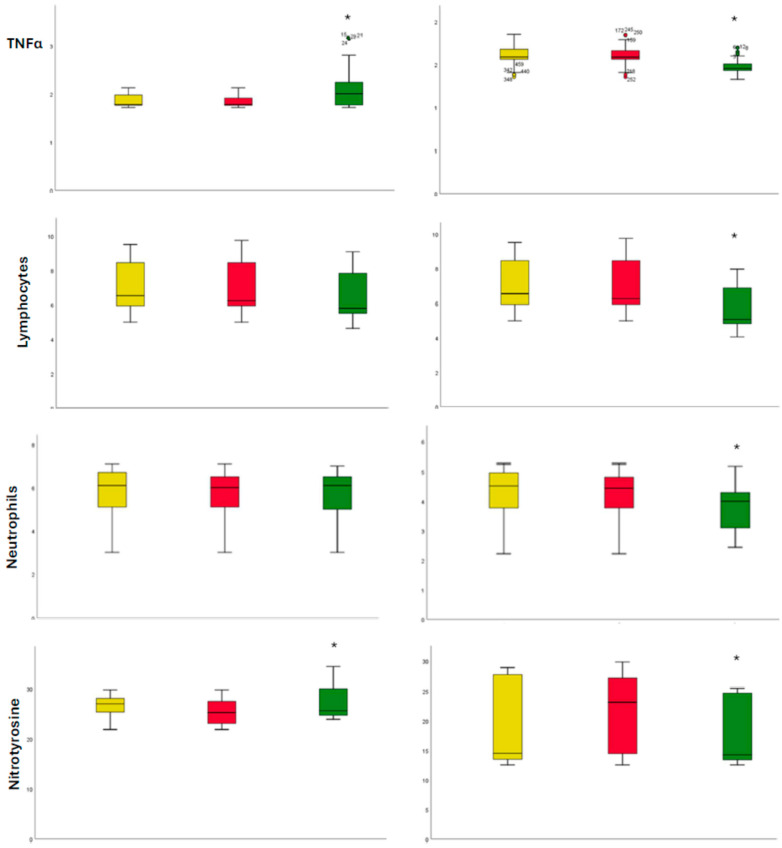
Bar graphs of serum levels of expression of inflammatory molecular and cellular markers, at baseline (left part of image) and follow-up end (right part of image). Yellow color: SGLT2i-users; red color: GLP-1RAs users; green color: SGLT2i/GLP-1RAs users. The values are expressed in pg/mL. CRP: c reactive protein; IL 6: interleukin 6; TNFα: tumor necrosis factor alpha. *: statistically significant (*p* < 0.05) vs. SGLT2i/GLP-1RAs combined therapy group of patients. The shapes are derived from blots images.

Kaplan–Meier curves show a statistically significant reduction in the composite endpoint in the combined therapy cohort compared to SGLT2i-users and GLP1RAs-users (*p* < 0.05) (Figure 1). This suggests a potential synergistic antiarrhythmic effect of combined therapy, even in patients with suboptimal glycemic control. The rate of any single event for a composite outcome is reported in Figure 1. The Cox regression analysis at 365 days of follow-up in the study population revealed that AF recurrence was predicted by BNP (HR 1.040, CI 95% 1.001–1.080), CRP (HR 1.223, CI 95% 1.143–1.309), and combined SGLT2i/GLP-1RAs therapy (HR 0.443, CI 95% 0.231–0.847), (*p* < 0.05) (Table 2). The patients under combined GLP-1RAs/SGLT-2i therapy, as compared to SGLT2i-users and GLP-1RAs-users, had significantly higher expressions of serum inflammatory and oxidative stress molecular markers at baseline (*p* < 0.05) (Figure 2). At the follow-up end, despite worse glycemic control, the patients under combined therapy with GLP-1RAs/SGLT2i, as compared to patients in monotherapy (SGLT2i-users and GLP-1RAs), had significantly lower expression of inflammatory cellular and molecular markers, as well as nytrotirosine (*p* < 0.05) (Figure 2).

## 3. Discussion

In this prospective cohort study, we found that combined GLP-1RAs/SGLT-2i therapy was associated with a significant reduction in AF recurrence after catheter ablation in T2DM patients, despite worse baseline and follow-up glycemic control. Higher values of BNP and CRP increased the risk of having AF at 1 year of follow-up post-catheter ablation. Conversely, the combined SGLT2i/GLP-1RAs reduced by about 56% the risk of having AF recurrence post-catheter ablation at 1 year of follow-up. Therefore, the combined SGLT2i/GLP-1RAs therapy results in greater reductions in inflammatory and oxidative stress markers as compared to monotherapy with SGLT2i or GLP-1RAs, suggesting that the observed antiarrhythmic benefit may be mediated by mechanisms beyond glucose-lowering. Intriguingly, these findings support the hypothesis that the complementary actions of SGLT2i and GLP-1RAs, via the improvement in myocardial substrate and reduction in inflammatory/oxidative stress, may synergistically contribute to a more favorable atrial remodeling process and sustained sinus rhythm post-ablation. Notably, the reduction in AF recurrence occurred even in a population with suboptimal HbA1c, underscoring the potential role of these agents as disease-modifying therapies rather than merely hypoglycemic drugs. In this setting, the BNP and CRP, as markers of advanced atrial remodeling and inflammation, could both be implied in a higher risk of AF recurrence, as reported in recent studies [7,8]. Indeed, the over-inflammation/oxidative stress is a main cause of atrial electrical and mechanical remodeling and AF recurrence post-catheter ablation [8]. Furthermore, our study results align with recent evidence showing that both SGLT2i [9,10,11] and GLP-1RAs [12] independently reduce AF recurrence after ablation and improve cardiovascular outcomes in T2DM cohorts. The additive benefit observed with combination therapy highlights a promising therapeutic strategy for high-risk diabetic patients undergoing AF ablation. Nevertheless, these findings should be interpreted in light of the study’s observational design and the relatively limited sample size of the dual therapy group. Randomized controlled trials are warranted to validate these preliminary results and to clarify the underlying pathophysiological pathways.

## 4. Materials and Methods (Research Design and Methods)

This is a prospective study involving 3 cohorts of T2DM patients who underwent catheter ablation for AF between January 2017 and June 2024. AF ablation was performed using either RF or pulsed field ablation PFA, with no procedures performed using cryoballoon technology. The distribution of ablation modality was comparable among groups

Eligible participants were ≥18 years with a confirmed diagnosis of T2DM and documented paroxysmal AF and indication to receive catheter ablation as recommended [13]. T2DM patients with glycated hemoglobin (HbA1c) < 7% were considered in good glycemic control, and thus no drug was added to their glucose-lowering chronic (at least 3 months) regimen and remained either on SGLT2i (SGLT2i-users, n 220) or GLP-1RAs (GLP-1RAs-users, n 193), [14]. The T2DM patients with poor glycemic control (HbA1c ≥ 7%) at study admission were prescribed either an SGLT2i or a GLP-1RAs to receive a GLP-1RAs/SGLT-2i combination therapy (GLP-1RAs/SGLT2i-users, n 69) [7], after AF ablation, the study population respected the following inclusion and exclusion criteria:

**Inclusion criteria**: at least 18 years of age, with a clinical history of paroxysmal AF with indication to receive catheter ablation; T2DM under chronic GLP1-RA and/or SGLT2i therapy.

**Exclusion criteria**: age < 18 or >75 years, contraindication to receive AF ablation, hyperkalemia, systolic hypotension (systolic blood pressure < 90 mmHg); patients with an estimated glomerular filtration rate (eGFR) < 30 mL per minute per 1.73 m^2^ of the body surface area; absence of informed patient consent, and any condition that would make survival for one year unlikely.

All patients were monitored for 1-year post-ablation for AF recurrence using ICM. The patients received an ICM 2 ± 4 months before ablation. Patient demographics, comorbidities, and medication use (including SGLT2i, GLP-1Ras, etc.), baseline HbA1c levels, and echocardiographic parameters were extracted from electronic medical records. Procedural data, including ablation technique, duration, and periprocedural complications were also recorded. The main composite outcome was cardioversion, new class 1 or 3 antiarrhythmic drug (AAD) use, redo ablation after a 3-month blanking period after the index AF ablation and AF recurrence (first ICM-detected AF ≥ 30 s event). The secondary outcome was the inflammatory/oxidative stress burden in the study cohorts at follow-up. The study was approved by the Ethical Committee of Campania 2, University of Campania “Luigi Vanvitelli”, with approval code 17.2020, in June 2020. The patients enrolled in the study signed and approved the consent for the study participation.

### 4.1. Laboratory Analysis

We evaluated, at baseline and after a 12-month follow-up, the plasma levels of glucose, Hb1Ac, and serum lipids, by enzymatic assays after an overnight fast. We evaluated the inflammatory burden, at baseline and after a 12-month follow-up, by the serum levels of pro-inflammatory cytokines (tumor necrosis alpha, TNFα, interleukin-6, IL-6), systemic inflammatory markers (C reactive protein, CRP), leucocytes, and neutrophils count. We used commercially available enzyme-linked immunosorbent assays (ELISAs) kits for the determination of TNFα, IL-6, and CRP (TNFα: TNF alpha Human ELISA Kit KHC3011, ThermoFisher Scientific, MA, USA; Human IL-6 Quantikine ELISA Kit D6050, R&D Systems, MN, USA; CRP: CRP Human ELISA kit KHA0031, ThermoFisher Scientific, MA, USA). We used an ice-cooled blood collection system to collect blood samples, immediately centrifuged for 10 min at 2.500 rpm at 4 °C, and then we isolated the supernatants containing serum samples and stored them at −80 °C before proceeding with ELISAs.

### 4.2. Statistical Analysis

We performed statistical analyses with SPSS Statistics version 22 (IBM Corp., Armonk, NY, USA). We presented the continuous variables as mean ± standard deviation when normally distributed or as median [interquartile range] if skewed; the categorical data were summarized as counts and percentages. We assessed the distribution of each biomarker at baseline and at 12 months by the Shapiro–Wilk test. Variables exhibiting significant skewness (*p* < 0.05) were log-transformed prior to parametric testing, with non-parametric alternatives employed if normalization was unsuccessful.

We evaluated the changes within each treatment cohort (SGLT2-i, GLP-1RAs, and combination therapy) from baseline to 1 year using paired t-tests for normally distributed data or Wilcoxon signed-rank tests for non-normal data. Between-group differences at baseline were examined by one-way ANOVA with Tukey’s post hoc adjustment or by Kruskal–Wallis tests with Dunn’s correction, and differential treatment effects over time were explored via repeated measures ANOVA (Greenhouse–Geisser correction) including “time,” “treatment group,” and their interaction. Then, we proved the significant interactions with Bonferroni-adjusted pairwise contrasts. Associations among biomarker changes were quantified using Pearson’s correlation for normally distributed differences or Spearman’s ρ otherwise. Kaplan–Meier survival analysis was used to estimate risk of AF recurrence. Cox proportional hazards models were employed to identify predictors of recurrence, adjusting for potential confounders. All tests were two-tailed, and statistical significance was defined as *p* < 0.05, except where adjusted thresholds applied for multiple comparisons.

## 5. Conclusions

Combined therapy with SGLT2i and GLP-1RAs could reduce about 56% the AF recurrence at 1 year of follow-up post-catheter ablation. These effects are linked to the significant reduction in inflammatory cellular and molecular markers despite worse periprocedural and follow-up term glycemic control. The anti-inflammatory and cardioprotective properties (reduction in AF recurrence) could be due to the synergistic anti-inflammatory and cardioprotective effect of SGLT2i [9,10,11] added to GLP-1RAs therapy [12] in T2DM with paroxysmal AF undergoing catheter ablation, beyond glycemic control. These synergistic effects played by SGLT2i and GLP-1RAs could improve post-ablation outcomes in high-risk diabetic patients. Further randomized trials are warranted to confirm these observations and explore the mechanistic pathways underlying this therapeutic benefit.

## Figures and Tables

**Table 1:** Clinical characteristics of study cohorts at baseline and at follow-up end. The study population included SGLT2i-users (n 220), GLP-1RAs users (n 193), and combined SGLT2i/GLP-1RAs therapy (n 69). ACEi: angiotensin converting enzyme inhibitors; ARBs: angiotensin receptors blockers; ARNI: angiotensin receptor neprilysin inhibitors; BNP: B type natriuretic peptide; CKD: chronic kidney disease; eGFR: estimated glomerular filtration rate; GLP-1RAs: Glucagon-Like Peptide-1 Receptor Agonists; HbA1c: glycated hemoglobin 1 Ac; LDL: low-density lipoprotein; LVEF: left ventricle ejection fraction; SGLT2i: sodium–glucose transporter 2 inhibitors. *: statistically significant (*p* < 0.05) vs. SGLT2i/GLP-1RAs cohort of patients. The symbol “/” is for: it does not change, as same percentage of male, white and black or African American.

Baseline				1 Year of Follow-Up		
Demographics	SGLT2i (n 220)	GLP1-RAs (n 193)	GLP1-RAs/SGLT2i (n 69)	SGLT2i (n 220)	GLP1-RAs (n 193)	GLP1-RAs/SGLT2i (n 69)
Age (years)	53.9 ± 8.4	54.5 ± 8.1	53.8 ± 8.0	/	/	/
Male, n (%)	142 (64.5)	119 (61.7)	42 (60.9)	/	/	/
White	210 (95.4)	182 (94.3)	66 (95.6)	/	/	/
Black or African American	10 (4.5)	11 (5.7)	3 (4.4)	/	/	/
Body Mass Index (kg/m^2^)	26.6 ± 5.95	26.1 ± 5.62	25.5 ± 5.44	26.4 ± 5.47	26.0 ±5.58	25.2 ± 5.3
Waist to hip ratio	0.89 ± 0.06	0.88 ± 0.07	0.88 ± 0.05	0.91 ± 0.07	0.90 ± 0.07	0.90 ± 0.06
Heart rate, bpm	75 ± 7	76 ± 6	77 ± 7	73 ± 6	74 ± 7	70 ± 7 *
**Comorbidities**						
Hypertension, n (%)	124 (56.4)	127 (65.8)	41 (59.4)	130 (59.1)	129 (66.8)	47 (68.1)
Dyslipidemia, n (%)	77 (35%)	83 (43.0)	38 (55.1)	99 (45)	103 (53.4)	41 (59.4)
Ischemic heart disease, n (%)	130 (59.1)	120 (62.2)	44 (63.8)	137 (62.3)	127 (65.8)	45 (65.2)
Thyroid disorder, n (%) disorders	45 (20.4)	37 (19.2)	15 (21.7)	49 (22.3)	45 (23.3)	17 (24.6)
CKD stage 3	23 (10.5)	18 (9.3)	7 (10.1)	27 (12.3)	29 (15.0)	9 (13.0)
CKD stage 4	5 (2.3)	4 (2.1)	1 (1.5)	6 (2.7)	5 (2.6)	2 (2.9)
**Laboratory values**						
HbA1c (%)	6.5 ± 0.3	6.6 ± 0.3	8.0 ± 0.4 *	6.4 ± 0.3	6.5 ± 0.3	7.2 ± 0.4 *
Glycemia, mg/dL	140 ± 25	142 ± 27	155 ± 30 *	130 ± 22	132 ± 23	145 ± 28 *
eGFR, mL/min/1.73 m^2^	68.9 ± 21.3	70.8 ± 20.8	61.6 ± 21.7 *	76.8 ± 27.6	72.4 ± 28.1	72.3 ± 33.3
LDL, mg/dL	72.4 ± 21.4	74.1 ± 21.0	76.2 ± 18.1	65.9 ± 19.4	67.3 ± 19.0	69.4 ± 16.3
BNP, pg/mL	66.9 ± 46.5	68.2 ± 48.3	70.1 ± 70.1	56.3 ± 38.1	58.5 ± 39.7	53.3 ± 39.6
LVEF (%)	55.1 ± 5.2	55.5 ± 5.0	54.2 ± 5.2	55.8 ± 4.8	56.4 ± 5.4	56.4 ± 5.2
left atrial volume, mL	35.5 ± 8.4	35.7 ± 8.5	36.2 ± 9.6	34.4 ± 7.8	34.1 ± 7.3	34.3 ± 6.8
**Drug therapy**						
Amiodaron, n (%)	29 (13.2)	23 (11.9)	10 (14.5)	22 (10)	19 (9.8)	6 (8.7)
Flecainide, n (%)	162 (73.6)	149 (77.2)	54 (78.3)	109 (49.5)	101 (52.3)	24 (34.8) *
Beta blockers, n (%)	153 (69.5)	144 (74.6)	48 (69.6)	162 (73.6)	150 (77.7)	51 (73.9)
Calcium blockers, n (%)	12 (5.5)	9 (4.8)	3 (4.3)	19 (8.6)	18 (9.6)	5 (7.2)
Anticoagulants, n (%)	198 (90)	181 (93.8)	63 (91.3)	205 (93.2)	184 (95.3)	65 (94.2)
Anti-platelets, n (%)	98 (44.5)	90 (46.6)	34 (49.3)	106 (48.2)	94 (48.7)	37 (53.6)
Metformin, n (%)	119 (54.1)	99 (51.3)	40 (57.9)	134 (60.9)	112 (58)	44 (63.8)
Insulin, n (%)	75 (34.1)	57 (29.5)	24 (34.8)	84 (38.2)	64 (33.2)	28 (40.6)
Digitalis, n (%)	43 (19.5)	40 (20.7)	16 (23.2)	49 (22.3)	46 (23.8)	21 (30.4)
ACEi, n (%)	51 (23.2)	50 (25.9)	12 (17.4)	55 (25)	54 (28)	16 (23.2)
ARBs, n (%)	63 (28.6)	62 (32.1)	24 (34.8)	74 (33.6)	72 (37.3)	27 (39.1)
ARNI, n (%)	17 (7.7)	18 (9.3)	8 (11.6)	25 (11.4)	26 (13.4)	10 (14.4)
Dihydropyridines, n (%)	22 (10)	19 (9.8)	7 (10.1)	29 (13.2)	28 (14.5)	9 (13.0)
Loop diuretics, n (%)	81 (36.8)	75 (38.9)	30 (435.5)	89 (40.5)	90 (46.6)	30 (43.5)
Thiazides, n (%)	34 (15.5)	32 (16.6)	15 (21.7)	40 (18.2)	37 (19.2)	16 (23.2)

**Table 2:** Univariate and Multivariate Cox regression analysis for study population at 1 year of follow-up calculated for the risk of having atrial fibrillation (AF) recurrence.

Univariate Analysis				Multivariate Analysis		
Risk Factor	HR	CI 95%	*p* Value	HR	CI 95%	*p* Value
Age	1.001	0.990–1.012	0.897	0.999	0.986–1.012	0.887
BNP	1.109	1.060–1.220	0.040 *	1.040	1.001–1.080	0.006 *
CRP	1.451	1.176–1.734	0.038 *	1.223	1.143–1.309	0.001 *
eGFR	1.006	0.999–1.014	0.095	0.999	0.990–1.008	0.795
Flecainide	0.884	0.614–1.273	0.507	1.118	0.729–1.714	0.610
Gender	1.154	0.825–1.616	0.402	1.014	0.696–1.477	0.943
Hb1Ac	1.154	1.074–1.441	0.050 *	1.010	0.909–1.122	0.857
Heart rate	0.997	0.974–1.022	0.831	1.016	0.988–1.046	0.267
Hypertension	0.681	0.494–0.938	0.019 *	0.934	0.619–1.410	0.747
Lymphocytes	1.387	0.733–1.956	0.090	1.086	0.931–1.266	0.296
LVEF	0.993	0.979–1.007	0.324	0.994	0.978–1.010	0.428
SGLT2i/GLP-1RAs	0.504	0.285–0.890	0.018 *	0.443	0.231–0.847	0.014 *

BNP: B type natriuretic peptide; CRP: C reactive protein; eGFR: estimated glomerular filtration rate; HbA1c: glycated hemoglobin 1 Ac; LVEF: left ventricle ejection fraction; SGLT2i/GLP-1RAs: combined therapy with sodium–glucose transporter 2 inhibitors and Glucagon-Like Peptide-1 Receptor Agonists; *: statistically significant (*p* < 0.05).

## Data Availability

The data that support the findings of this study are available from the corresponding author upon reasonable request.
